# Heterologous reconstitution of the biosynthesis pathway for 4-demethyl-premithramycinone, the aglycon of antitumor polyketide mithramycin

**DOI:** 10.1186/s12934-020-01368-3

**Published:** 2020-05-24

**Authors:** Daniel Zabala, Lijiang Song, Yousef Dashti, Gregory L. Challis, José A. Salas, Carmen Méndez

**Affiliations:** 1 Departamento de Biología Funcional e Instituto Universitario de Oncología del Principado de Asturias (I.U.O.P.A), University of Oviedo, Oviedo, Spain; 2 Instituto de Investigación Sanitaria de Asturias (ISPA), Oviedo, Spain; 3 Department of Chemistry, University of Warwick, Coventry, CV4 7AL UK; 4 Warwick Integrative Synthetic Biology Centre, University of Warwick, Coventry, CV4 7AL UK; 5 Department of Biochemistry and Molecular Biology, Monash University, Clayton, VIC 3800 Australia

**Keywords:** *Streptomyces argillaceus*, Mithramycin, Premithramycinone, Aureolic acid, Acyl-CoA ligase, Cyclase, Polyketide synthase

## Abstract

**Background:**

Mithramycin is an anti-tumor compound of the aureolic acid family produced by *Streptomyces argillaceus*. Its biosynthesis gene cluster has been cloned and characterized, and several new analogs with improved pharmacological properties have been generated through combinatorial biosynthesis. To further study these compounds as potential new anticancer drugs requires their production yields to be improved significantly. The biosynthesis of mithramycin proceeds through the formation of the key intermediate 4-demethyl-premithramycinone. Extensive studies have characterized the biosynthesis pathway from this intermediate to mithramycin. However, the biosynthesis pathway for 4-demethyl-premithramycinone remains unclear.

**Results:**

Expression of cosmid cosAR7, containing a set of mithramycin biosynthesis genes, in *Streptomyces albus* resulted in the production of 4-demethyl-premithramycinone, delimiting genes required for its biosynthesis. Inactivation of *mtmL*, encoding an ATP-dependent acyl-CoA ligase, led to the accumulation of the tricyclic intermediate 2-hydroxy-nogalonic acid, proving its essential role in the formation of the fourth ring of 4-demethyl-premithramycinone. Expression of different sets of mithramycin biosynthesis genes as cassettes in *S. albus* and analysis of the resulting metabolites, allowed the reconstitution of the biosynthesis pathway for 4-demethyl-premithramycinone, assigning gene functions and establishing the order of biosynthetic steps.

**Conclusions:**

We established the biosynthesis pathway for 4-demethyl-premithramycinone, and identified the minimal set of genes required for its assembly. We propose that the biosynthesis starts with the formation of a linear decaketide by the minimal polyketide synthase MtmPKS. Then, the cyclase/aromatase MtmQ catalyzes the cyclization of the first ring (C7–C12), followed by formation of the second and third rings (C5–C14; C3–C16) catalyzed by the cyclase MtmY. Formation of the fourth ring (C1–C18) requires MtmL and MtmX. Finally, further oxygenation and reduction is catalyzed by MtmOII and MtmTI/MtmTII respectively, to generate the final stable tetracyclic intermediate 4-demethyl-premithramycinone. Understanding the biosynthesis of this compound affords enhanced possibilities to generate new mithramycin analogs and improve their production titers for bioactivity investigation.

## Background

Mithramycin (MTM) is an anti-tumor agent member of the aureolic acid family and is produced by *Streptomyces argillaceus* ATCC12956. MTM was approved as an anticancer drug in 1970 [1], but its use has been limited due to its toxic side effects. MTM acts as an anti-tumor agent by binding to G/C rich regions located at gene promoters (Sp1 or Sp3), preventing the binding of transcription factors and thus blocking the action of Sp regulated genes [2]. In the past few years, new activities and other potential uses for MTM have been discovered, reviving interest in this compound [3–7]. In addition, new formulations for MTM have been developed (i.e. MTM-loaded nanoparticles) [8], and a MTM phase I/II trial was performed with children and adults with refractory Ewing sarcoma [9].

Structurally, MTM is a polyketide (PK) consisting of a tricyclic aglycone with two aliphatic side chains, which is glycosylated by a trisaccharide and a disaccharide [10]. The MTM biosynthesis gene cluster (BGC) *mtm* has been cloned and characterized [11]. This has allowed the generation of new MTM analogs through combinatorial biosynthesis strategies [12]. Some of these new analogs showed higher antitumor activity and/or lower toxicity than the parental compound [13–16], and were shown to affect the Ewing sarcoma xenograft growth and triple negative breast cancer growth in mice [17, 18]. These promising activities have led to the development of these compounds as potential new anticancer drugs [19, 20], which requires sufficient amounts for preclinical and clinical trials.

Different approaches have been used to improve production of MTM and some analogs. These include overexpression of cluster specific regulators [21, 22], increasing and channeling the flux of MTM precursors using metabolic engineering strategies [23], and heterologous expression of the *mtm* BGC [24]. Further improvements could be achieved using synthetic biology techniques [25]. This approach requires the identification of all genes involved in the biosynthesis of a compound and determination of the order in which the gene products act. The biosynthesis pathway of MTM proceeds through the formation of the tetracyclic aglycon 4-demethyl-premithramycinone (4DMPC). This is methylated and glycosylated to yield the tetracyclic intermediate premithramycin B, which undergoes oxidative cleavage at its fourth ring and a reduction of the resultant side chain to generate the final bioactive compound MTM [11]. All biosynthesis steps from 4DMPC to MTM have been identified and characterized through the generation of mutants and characterization of the accumulated compounds, by in vitro assays and/or by gene expression experiments [11, 12]. However, the biosynthetic steps leading to 4DMPC are not completely understood. Here we report the in vivo reconstitution of the biosynthesis pathway for 4DMPC, identifying the minimum set of genes required and the order of action of the gene products.

## Results and discussion

### Delimiting the set of genes required for 4-demethyl-premithramycinone biosynthesis

**Fig. 1:**
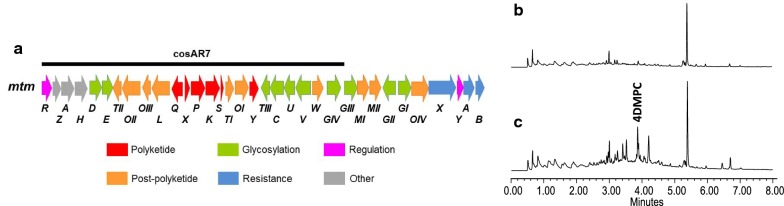
Expression of cosAR7 in *S. albus*. **a** Genetic organization of cluster *mtm* from *Streptomyces argillaceus*. **b** UHPLC analysis at 280 nm of organic extracts of *S. albus* pKC505 (control). **c** UHPLC analysis at 280 nm of organic extracts of *S. albus* cosAR7-R. Black line indicates *mtm* genes contained in cosmid cosAR7. Chromatograms are shown at the same scale. **4DMPC**, 4-demethyl-premithramycinone

The *mtm* BGC contains thirty-four genes (Fig. 1a), some of which have been associated with the biosynthesis of 4DMPC [11, 12]: *mtmP* (β-ketoacyl synthase α), *mtmK* (β-ketoacyl synthase β) and *mtmS* (acyl carrier protein), encoding the minimal polyketide synthase (PKS) [26, 27], the aromatase/cyclase MtmQ, the cyclase MtmY [28], and the oxygenase MtmOII [29–31]. Cosmid cosAR7 [27] (Fig. 1a) contains all these genes (Table 1), as well as some other genes proposed to be involved in the biosynthesis of 4DMPC, such as *mtmX* and *mtmTI* and *mtmTII,* which encode a putative cyclase and two ketoreductases, respectively. It also includes genes that have been shown to be dispensable for MTM biosynthesis in *S. argillaceus*: *mtmOI* and *mtmOIII* [31] and *mtmZ, mtmA, mtmH* (Fernández-Lozano, unpublished results); genes for deoxysugar biosynthesis (*mtmD, mtmE, mtmTIII, mtmC, mtmU* and *mtmV*) [32, 33], and transfer (*mtmGIV*) [34], and a ketoreductase (*mtmW*) involved in side chain reduction [35]. In addition, it contains *mtmL*, which encodes a putative acyl-CoA ligase. Therefore cosAR7 would have the potential capability to direct the biosynthesis of 4DMPC but not any other later biosynthesis intermediate since this cosmid lacks *mtmMI* [36], which encodes the methyltransferase MtmMI that catalyzes an essential step in MTM biosynthesis that takes place before glycosylation and further reduction steps. To confirm the potentiality of cosAR7, it was introduced into *Streptomyces albus* to generate the recombinant strain *S. albus* cosAR7 (Table 2). To ensure the correct expression of the *mtm* genes, plasmid pFL3R [22] expressing the pathway specific transcriptional activator *mtmR* was also introduced into *S. albus* cosAR7. As can be seen in Fig. 1c, the resultant strain (*S. albus* cosAR7-R; Table 2) was able to produce a compound with exactly the same absorptium spectrum and UHPLC retention time as the previously isolated mithramycin intermediate 4-DMPC [37]. This confirms that cosmid cosAR7 contains all the genes required for the biosynthesis of 4DMPC, and excludes the involvement of other *mtm* genes in the biosynthesis of this biosynthesis intermediate.

**Table 1:** Functions of gene products from cosAR7

Gene	aa	Proposed function	Similar protein (acc. number)	Identical aa (%)
*mtmZ*	260	Putative thioesterase	WP_164185915.1	84
*mtmA*	460	Methylenetetrahydrofolate reductase	WP_043385055.1	83
*mtmH*	482	Adenosylhomocysteinase	WP_164185914.1	94
*mtmD*	355	Glucose-1P thymidylyltransferase	WP_164189839.1	88
*mtmE*	331	dTDP-glucose 4,6-dehydratase	WP_164189984.1	91
*mtmTII*	253	Ketoreductase	WP_164189846.1	90
*mtmOII*	531	Oxygenase	WP_078655152.1	84
*mtmOIII*	99	Monooxygenase	WP_164189850.1	87
*mtmL*	514	Acyl-CoA ligase	WP_051703560.1	80
*mtmQ*	315	Aromatase	WP_164189988.1	89
*mtmX*	150	Putative cyclase	WP_030997656.1	81
*mtmP*	422	beta-ketoacyl-(ACP) synthase	WP_030997655.1	93
*mtmK*	408	Ketosynthase chain-length factor	WP_164189853.1	88
*mtmS*	85	Acyl carrier protein	WP_164189855.1	82
*mtmTI*	254	Ketoreductase	WP_164189991.1	86
*mtmOI*	436	FAD-binding monooxygenase	WP_164189993.1	86
*mtmY*	257	Cyclase	WP_164189857.1	91
*mtmTIII*	247	Ketoreductase	WP_164189858.1	89
*mtmC*	420	D-mycarose 3-C-methyltransferase	WP_164189995.1	91
*mtmU*	328	D-oliose 4-ketoreductase	WP_030997647.1	80
*mtmV*	486	NDP-hexose 2,3-dehydratase	WP_164189860.1	84
*mtmW*	326	Aldo/keto reductase	WP_164189862.1	89
*mtmGIV*	407	Glycosyltransferase	WP_164189864.1	89

**Table 2:** Plasmids and *Streptomyces argillaceus* and *Streptomyces albus* strains generated in this work

Plasmid	Expressed gene(s)	Vector used
pDZL10	*mtmL*	pEM4T
pDZPKS1	*mtmPKS*	pEM4
pDZPKSQ	*mtmPKSQ*	pEM4
pDZPKS2	*mtmPKSL*	pEM4
pDZPKS3	*mtmPKSLQ*	pEM4
pDZPKS4	*mtmPKSLQX*	pEM4
pDZPKS5	*mtmPKSLQXY*	pEM4
pDZPKS6	*mtmPKSLQXYOII*	pEM4
pDZPKS7	*mtmPKSLQXYOIITI*	pEM4
pDZPKS8	*mtmPKSLQY*	pEM4
pDZPKS9	*mtmPKSQY*	pEM4
pDZPKS10	*mtmPKSQYTI*	pEM4
pDZPKS11	*mtmPKSQYX*	pEM4
pDZPKS12	*mtmPKSQXYOIITITII*	pEM4
pDZPKS13	*mtmPKSQYXOIITII*	pEM4
pDZPKS14	*mtmPKSLQXYOIITITII*	pEM4
pDZPKS15	*mtmOII*	pEM4AT
pDZPKS19	*mtmOIITI*	pEM4AT
pDZPKS21	*mtmOIITII*	pEM4AT

Recombinant/mutant strain	Inactivated/expressed gene(s)	Plasmid used
*S. argillaceus* ΔL	*mtmL*	p∆L
*S. argillaceus* ΔL-pDZL10	*mtmL*	pDZL10
*S. argillaceus* pDZL10	*mtmL*	pDZL10
*S. argillaceus* pEM4T	–	pEM4T
*S. albus* cosAR7	*mtmZAHDETIIOIIOIIILQXPKSTIOIYTIIICUVWGIV*	cosAR7
*S. albus* cosAR7-R	*mtmZAHDETIIOIIOIIILQXPKSTIOIYTIIICUVWGIV*	cosAR7
	*mtmR*	pFL3R
*S. albus* pEM4	*–*	pEM4
*S. albus* pDZPKS1	*mtmPKS*	pEM4
*S. albus* pDZPKSQ	*mtmPKSQ*	pEM4
*S. albus* pDZPKS9	*mtmPKSQY*	pEM4
*S. albus* pDZPKS10	*mtmPKSQYTI*	pEM4
*S. albus* pDZPKS11	*mtmPKSQYX*	pEM4
*S. albus* pDZPKS12	*mtmPKSQXYOIITITII*	pEM4
*S. albus* pDZPKS13	*mtmPKSQYXOIITII*	pEM4
*S. albus* pDZPKS8	*mtmPKSLQY*	pEM4
*S. albus* pDZPKS5	*mtmPKSLQXY*	pEM4
*S. albus* pDZPKS5+15	*mtmPKSLQXY*	pEM4
	*mtmOII*	pEM4AT
*S. albus* pDZPKS8+15	*mtmPKSLQY*	pEM4
	*mtmOII*	pEM4AT
*S. albus* pDZPKS5+21	*mtmPKSLQXY*	pEM4
	*mtmTIIOIITI*	pEM4AT

### Role of *mtmL* in mithramycin biosynthesis

**Fig. 2:**
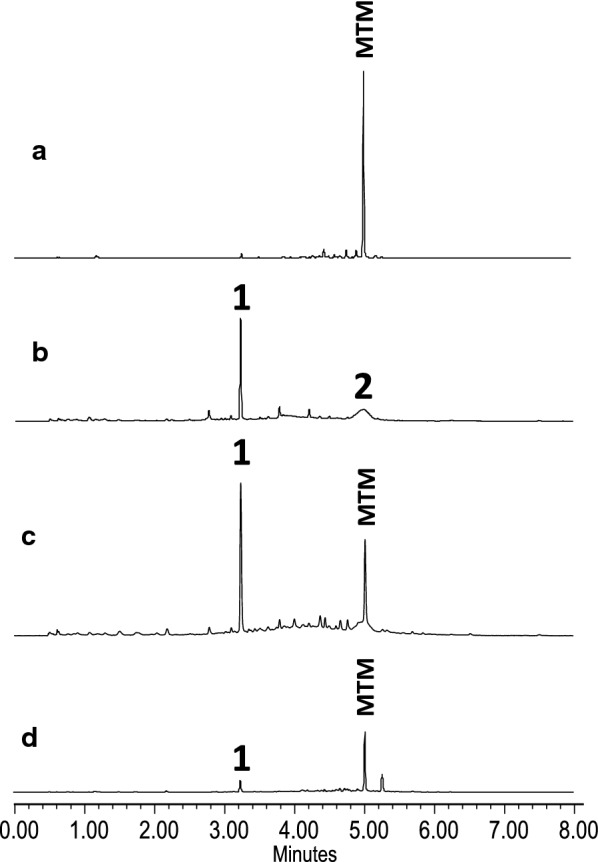
UHPLC chromatograms at 280 nm of organic extracts of *S. argillaceus* strains. **a***S. argillaceus* wild type strain (WT); **b***S. argillaceus* ΔL; **c***S. argillaceus* ΔL-pDZL10; **d***S. argillaceus* ΔL fed with premithramycinone. Chromatograms are shown at the same scale. **1,** SEK15; **2,** 2-hydroxy nogalonic acid; **MTM**, mithramycin

**Fig. 3:**
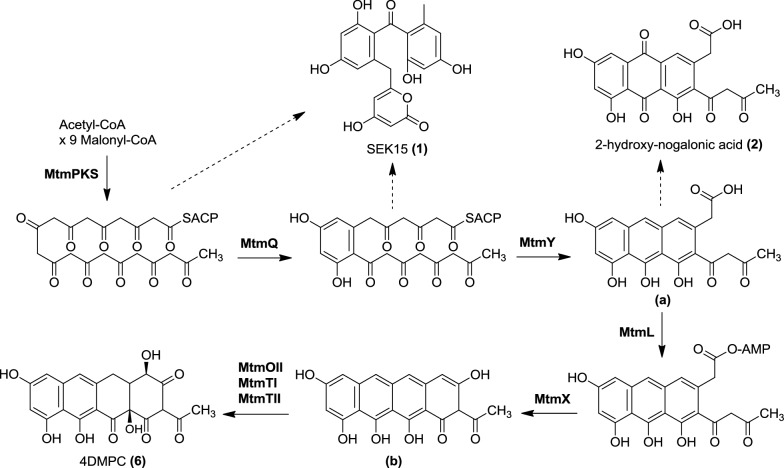
Proposed biosynthesis pathway for 4-demethyl-premithramycinone. Dashed lines represent formation of shunt products

CosAR7 contains *mtmL,* encoding a putative acyl-CoA ligase, whose role is unclear. Comparison of the MtmL sequence with those of proteins in databases showed similarity with acyl-CoA ligases such as CmmLII, PokL and SsfL2 (53%, 49% and 45% of identical aminoacids, respectively), encoded by the chromomycin A_3_, polyketomycin and tetracycline SF2575 BGCs, respectively [38–40] (Additional file 1: Figure S1) Based on the similarity of MtmL with other acyl-CoA ligases, it was initially proposed that it could play a role in supplying acetyl-CoA to the MTM pathway [31]. More recently, it has been found that some ATP-dependent acyl-CoA ligases such as SsfL2 and OxyH are involved in formation of the fourth ring of tetracyclines, and that MtmL could fulfill such a role when combined with tetracycline biosynthesis enzymes [41, 42]. To determine the role of *mtmL* in the MTM pathway, this gene was initially overexpressed in the *S. argillaceus* wild type strain using plasmid pDZL10 (Table 2 and Additional file 1). MTM production in the resultant recombinant strain (*S. argillaceus* pDZL10; Table 2) was 29% higher than in the control strain (*S. argillaceus* pEM4T) (Additional file 1: Figure S2). This result supported a role for MtmL in supplying precursors for the biosynthesis of the MTM aglycon. Then, *mtmL* was inactivated in *S. argillaceus* by replacing the wild type copy of this gene by a mutated one using plasmid pΔL (Table 2 and Additional file 1: Figure S3). This plasmid contains an apramycin resistance cassette (*aac(3)IV*) cloned into *mtmL* in the same direction of transcription to avoid a polar effect on downstream genes. The resultant mutant strain (*S. argillaceus* ΔL; Table 2) did not produce MTM, but two new metabolites were detected instead (Fig. 2b). The major compound showed an absorbance maximum at 289 nm and had an [M + H]^+^ ion with *m/z* = 385.0936, corresponding to the molecular formulae C_20_H_16_O_8_. Purification and structural characterization of this compound by NMR spectroscopy and MS (Additional file 2) showed it is the shunt product SEK15 (**1,** Fig. 3). This compound is generated by spontaneous cyclization of the 20-carbon polyketide chain assembled by the minimal PKS [26], and it was detected for the first time in *S. coelicolor* CH999, expressing the minimal PKS from the tetracenomycin biosynthesis pathway [43]. Production of this compound disproved the proposed role for MtmL in supplying acetyl-CoA units for the biosynthesis of the MTM polyketide chain. The second major compound exhibited an absorbance maximum at 290 nm and a molecular formulae of C_20_H_14_O_9_ was obtained from HR-ESI-MS (*m/z* calculated for [M + H]^+^: 399.0711; observed: 399.0710). This compound was purified and structurally characterized by NMR spectroscopy and MS (Additional file 3), which showed to be the tricyclic quinone 2-hydroxy nogalonic acid (**2,** Fig. 3). This compound has been proposed to be an intermediate in steffimycin biosynthesis and is produced in the absence of the fourth ring cyclase [44]. Thus, MtmL most probably is required for cyclization of the fourth ring of 4DMPC and acts prior to oxygenation of the aglycon.

To confirm that *S. argillaceus* ∆L was only affected in *mtmL*, this strain was complemented using plasmid pDZL10 (Table 2). As can be observed in Fig. 2c, MTM production was restored in the resulting strain (*S. argillaceus* ΔL-pDZL10; Table 2). Moreover, the mutant strain was chemically complemented by growing it in the presence of 50 µg/mL premithramycinone. This compound is a MTM biosynthesis intermediate, accumulated by the *S. argillaceus* M7G4 mutant that is affected in the *mtmGIV* glycosyltransferase gene [34]. In the MTM biosynthesis pathway, premithramycinone is generated by methylation of 4DMPC by the methyltransferase MtmMI [36]. Samples obtained after 48 h of incubation in the presence of premithramycinone showed its complete conversion into MTM (Fig. 2d). All these results confirm that blockage of MTM production in *S. argillaceus* ∆L is due solely to the mutation of *mtmL* and supports a role for MtmL in the biosynthesis of the MTM aglycon, after initial polyketide chain assembly. They also suggest that MtmL and/or its reaction product is limited within the pathway, since overexpression of *mtmL* in the wild type strain results in an increase in MTM production. Improvement of production yields by overexpression of structural genes has also been observed in other biosynthetic pathways [45].

### In vivo reconstitution of the 4-demethyl-premithramycinone biosynthetic pathway

**Fig. 4:**
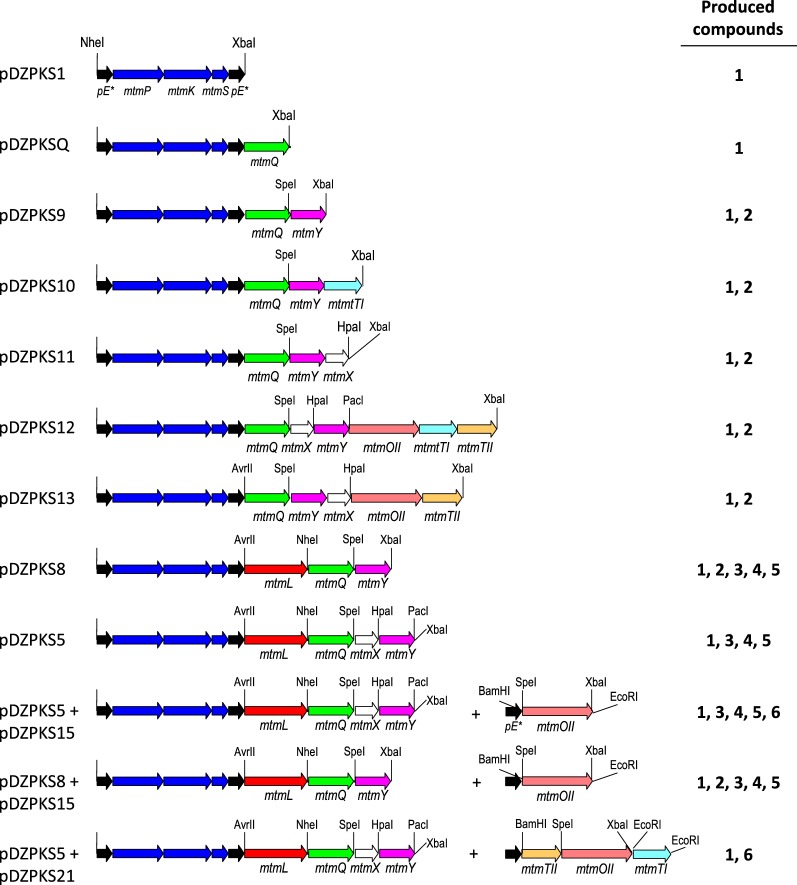
Genetic organization of *mtm* biosynthesis gene cassettes used to investigate 4-demethyl-premithramycinone biosynthesis. pE*, promoter for the erythromycin resistance gene (ermEp*); **1**, SEK15; **2**, 2-hydroxy nogalonic acid; **3** to **5**, unknown compounds; **6**, 4-demethyl-premithramycinone

**Fig. 5:**
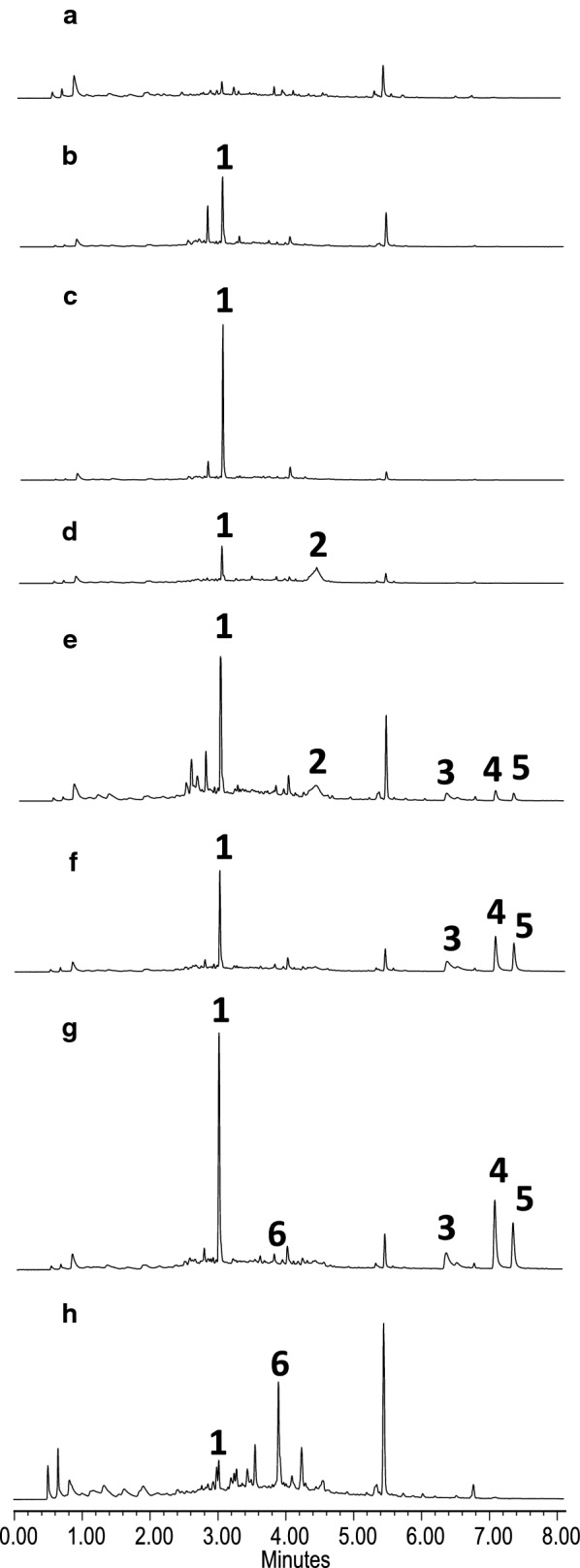
UHPLC analysis at 280 nm of organic extracts of *S. albus* strains expressing different cassette plasmids. **a***S. albus* pEM4 (control); **b***S. albus* pDZPKS1; **c***S. albus* pDZPKSQ; **d***S. albus* pDZPKS9; **e***S. albus* pDZPKS8; **f***S. albus* pDZPKS5; **g***S. albus* pDZPKS5 + 15; **h***S. albus* pDZPKS5 + 21. Chromatograms are shown at the same scale. **1,** SEK15; **2,** 2-hydroxy nogalonic acid; **3-5**, unknown compounds; **6**, 4-demethyl-premithramycinone

Based on the above results, we aimed to reconstitute the biosynthesis pathway for 4DMPC in vivo to determine the minimal set of genes required and the order of biosynthetic steps. To do this, we used gene cassettes flanked by unique restriction sites to create artificial operons under the control of the ermE* promoter containing different combinations of the *mtmQ*, *mtmX*, *mtmY*, *mtmOII*, *mtmTI* and *mtmTII* genes (Table 1) downstream from an operon containing the *mtmP*, *mtmK* and *mtmS* genes (Fig. 4, Table 2 and Additional file 4). The resulting plasmids were introduced into *S. albus*, and the metabolites produced by the resultant strains (Table 2) were compared using UHPLC analyses (Fig. 5).

The strain containing a construct (pDZPKS1) expressing just the *mtmP, mtmK* and *mtmS* genes, which encode the minimal PKS, produced the shunt metabolite SEK15 (**1**) that shows a C7-C12 cyclization (Table 2, Figs. 3 and 5b). This metabolite was absent from the control strain (*S. albus* pEM4; Table 2 and Fig. 5a). Production of SEK15 by *S. albus* pDZPKS1 agrees with reports that indicate that the minimal PKS is sufficient to determine the regioselective C7–C12 cyclisation [46]. MtmQ contains an N-terminal and C-terminal aromatase/cyclase domains (cd08861), and it shows similarity to putative non-reducing di-domain aromatases/cyclases [47], such as WP_164189988.1 and WP_030997657.1 (89% and 85% identical aminoacids, respectively) or StfQ from the steffimycin BGC [44] (53% identical aminoacids. MtmQ was proposed to be involved in the cyclisation between C7 and C12 [48], which was later on confirmed by expressing in *Escherichia coli mtmQ* together with an engineered fungal PKS that does not promote this type of cyclisation [49]. When *mtmQ* was co-expressed with *mtmPKS* using plasmid pDZPKSQ (Table 2 and Fig. 4) the metabolite profile did not change, but the titre of **1** increased about 25% (Fig. 5c). This increase can be explained by MtmQ cyclisating and/or facilitating and accelerating the proper cyclisation pattern of the polyketide chain, as it has been previously suggested [47].

MtmY shows similarity to cyclases involved in second and third ring formation in aromatic polyketide biosynthesis, such as KstD1 (71%), StfY (69%), DspY (68%) and OxyN (68%) from the kosinostatin, steffimycin, daunorubicin and oxytetracycline pathways, respectively [44, 50–52]. Co-expression of *mtmY* with *mtmPKS* and *mtmQ* (using pDZPKS9; Table 2 and Fig. 4) led to the production of 2-hydroxy nogalonic acid (**2**), in addition to **1** (Figs. 3 and 5d). This result supports the role of MtmY as a cyclase involved in formation of the second and third rings and agrees with results from in vitro assays using MtmY in combination with enzymes from other pathways [28]. Addition of the putative ketoreductases encoded by *mtmTI* and *mtmTII*, the oxygenase encoded by *mtmOII* and/or the cyclase encoded by *mtmX*, which is proposed to be involved in formation of the fourth ring [53] (using plasmids pDZPKS10, pDZPKS11, pDZPKS12 and pDZPKS13; Table 2 and Fig. 4) did not alter the product profiles. This result and the fact that *S. argillaceus* ∆L and *S. albus*-pDZPKS9 showed similar metabolite profiles (see Figs. 2b and 5d) suggests MtmL acts immediately after MtmY. Therefore, we co-expressed *mtmL* together with *mtmPKS*, *mtmQ* and *mtmY* (using plasmid pDZPKS8; Table 2 and Fig. 4). The resulting strain (*S. albus* pDZPKS8) had a modified metabolite profile (Fig. 5e): in addition to compounds **1** and **2**, this strain produced three new metabolites (compounds **3** to **5**), with retention times of 6.3, 7 and 7.3 min, respectively. Additional expression of *mtmX* (pDZPKS5; Table 2 and Fig. 4) did not alter the metabolite profile, but the titres of compounds **3**, **4** and **5** increased, whereas the production of compounds **1** and **2** was significantly reduced (Fig. 5f). Unfortunately, the low yields and instability of these compounds did not permit their purification and structural characterization, but the fact that they were only produced when *mtmL* (and *mtmX*) were co-expressed and they showed longer retention times than compound **2**, indicates they might be tetracyclic. These results confirm MtmL plays an essential role in the biosynthesis of the MTM aglycon and are consistent with its proposed role as cyclase involved in formation of the fourth ring [42]. MtmL likely adenylates the carboxyl group in the product of MtmY. The resulting acyl adenylate is activated towards cyclisation by attack of an enolate derived from deprotonation of the 1,3-diketone. We also suggest that MtmX and MtmL cooperate in this process, since expression of *mtmX* improved production of compounds **3** to **5**. MtmX is homologous to SnoaL-family of enzymes that have been shown to mediate fourth ring closure in the biosynthesis of other aromatic polyketides [54]. In addition, MtmX is similar to StfX (49% identical aminoacids) from the steffimycin biosynthesis pathway, which has been proposed to play a role in facilitating formation of the fourth ring [44] and has been recently suggested to be responsible, together with ketoreductase StfT, for cyclization between C2 and C19 of presteffimycinone [28]. Thus, we propose that, in the MTM pathway, cyclisation of the product of MtmL to form the fourth ring can occur spontaneously, but is inefficient and that MtmX catalyses the reaction. This accounts for the increase in the production of **3**, **4** and **5** when MtmX is co-expressed with MtmL. Previous studies on MTM biosynthesis showed that *mtmOII* is essential for 4DMPC production [29, 31]. Therefore, we co-expressed *mtmOII* (pDZPKS15; Table 2 and Fig. 4) in *S. albus* with pDZPKS5. The resulting strain (*S. albus* pDZPKS5 + 15; Table 2 and Fig. 4) produced a small amount of 4DMPC (compound **6** in Fig. 5g). Expression of *mtmOII* in the absence of *mtmX* (pDZPKS8 plus pDZPKS15; Table 2 and Fig. 4) did not lead to the production of 4DMPC, establishing that MtmX is required for 4DMPC biosynthesis and that MtmOII acts after MtmL and MtmX. Co-expression of *mtmTI* and *mtmTII* with *mtmOII* (pDZPKS21; Table 2 and Fig. 4) and pDZPKS5 caused an increase in 4DMPC (**6**) production and the disappearance of compounds **3** to **5** (Fig. 5h).

These results demonstrate that the minimal set of enzymes required for the biosynthesis of 4DMPC is the minimal PKS MtmPKS, the aromatase MtmQ, cyclases MtmY and MtmX, the acyl-CoA ligase MtmL, the oxygenase MtmOII, and ketoreductases MtmTI and MtmTII. They also show that oxygenases MtmOI and MtmOIII are not required for the biosynthesis of 4DMPC, despite early proposals to the contrary [29]. Based on these results, we propose a pathway for 4DMPC biosynthesis (Fig. 3). This is initiated by the condensation of one acetyl-CoA and nine malonyl-CoA units catalyzed by the type II minimal PKS MtmPKS to generate a decaketide that spontaneously cyclises to afford the shunt product SEK15 (**1**). Then, the aromatase/cyclase MtmQ, followed by the cyclase MtmY form the first (C7–C12), second (C5–C14) and third (C3–C16) rings, generating a putative intermediate **a**. Spontaneous oxidation of the second ring to a quinone, a process that has also been observed for other aromatic polyketides [50], results in the formation of 2-hydroxy-nogalonic acid (**2**). Next, MtmL adenylates the carboxyl group of compound **a** and collaborates with MtmX to form the fourth ring (between C1–C18), generating the putative intermediate **b**, and oxygenation by MtmOII, followed by reduction by MtmTI and MtmTII, affords 4DMPC (**6**). Oxygenation of intermediate **b** could involve its epoxidation followed by reductive opening of the epoxide, as previously proposed [29].

In this pathway, MtmL and MtmX form the fourth ring prior to the oxidation and reduction reactions. This hypothesis is supported by the following facts: (i) inactivation of *mtmL* in *S. argillaceus* (mutant ΔL) interrupts MTM biosynthesis and results in accumulation of 2-hydroxy-nogalonic acid (**2**); (ii) heterologous expression of *mtmPKSQY* leads to the formation of 2-hydroxy-nogalonic acid (**2**) and in the absence of *mtmL*, expression of *mtmX*, *mtmOII*, *mtmTI* and/or *mtmTII* does not modify the metabolite profile, indicating that MtmL acts before the products of these genes; (iii) Co-expression of *mtmL* with *mtmPKSQY* results in the production of new compounds **3**, **4** and **5**, the titres of which are increased by co-expression of *mtmX*; and (iv) in the absence of *mtmX*, co-expression of *mtmOII* and *mtmPKSQYL* does not lead to the production of new compounds, suggesting that MtmX is required for biosynthesis to proceed and acts before MtmOII.

## Conclusions

The pathway to 4DMPC, a key intermediate in MTM biosynthesis, has been established through in vivo reconstitution in *S. albus*, using a sequential expression approach employing *mtm* gene cassettes. This has allowed the assignment of gene functions and determination of the order of biosynthetic steps. Understanding the biosynthetic pathway for 4DMPC will pave the way for improving the titre of MTM and analogs via synthetic biology approaches, and will facilitate rational creation of novel derivatives by metabolic engineering strategies. In addition, the gene cassette approach we employed here could be used in mix and match experiments with genes from other aromatic polyketide biosynthetic pathway and to reconstitute the biosynthesis of other aromatic polyketide aglycons.

## Methods

### Strains and culture conditions

*S. argillaceus* ATCC12956 a mithramycin producer, was used for gene replacement experiments. *S. albus* J1074 [55] was used as a host for heterologous reconstitution of the 4DMPC biosynthesis pathway. Sporulation of strains was achieved by growing them for 7 days at 30 °C on plates containing medium A [56]. Preparation of *Streptomyces* protoplasts, transformation, and selection of transformants was carried out as described [55]. *Escherichia coli* ET12567 (pUB307) was used as donor for intergeneric conjugation [55]. *E. coli* DH10B (Invitrogen) was used as a host for subcloning. Metabolite production was carried out on R5A solid medium [56] for 8 days and three independent cultures were performed for each production experiment as previously described [23]. Bioconversion experiments were carried out using a two-step culture method as previously described [57], except that cultivation in R5A liquid medium was carried out for 24 h before 50 μg/ml of premithramycinone were added to the culture. Compounds produced were analyzed after further 48 h of incubation in the presence of that metabolite. When plasmid-containing clones were grown, the medium was supplemented with the appropriate antibiotics: 5 or 25 μg/ml thiostrepton for liquid and solid cultures, respectively; 100 μg/ml ampicillin; 25 μg/ml apramycin; 25 μg/ml kanamycin; 25 μg/ml chloramphenicol and 25 μg/ml nalidixic acid.

### DNA manipulation and plasmids

Plasmid DNA preparations, restriction endonuclease digestions, and other DNA manipulations were according to standard procedures for *Streptomyces* and for *E. coli* [55, 58]. PCR amplifications were carried out using a Platinum Pfx DNA Polymerase (Invitrogen) and performed in a thermocycler (MyCicler Thermal Cycler System; Bio-Rad). pCR-blunt (Invitrogen), pIJ2925 [55], pUK21 [59], pEM4T [60], pEM4AT [61], pEM4 [61], and pBSKT [22], were used for subcloning. Plasmid pEFBA [36] was used as a source of the apramycin resistance cassette. Cosmid cosAR7 [27] was used as a source of *S. argillaceus* DNA for 4DMPC production.

### Generation of cassette plasmids for 4DMPC production

Several genes from the *mtm* BGC were amplified and sequenced using the primers listed in Additional file 4: Table S3. Multicistronic cassettes were constructed using XbaI/SpeI/NheI/AvrII/HpaI/PacI compatible ends to facilitate several combinations in the vectors pEM4 and pEM4AT. Detailed description on plasmids generation is presented in Additional file 4, and are shown in Table 2 and Fig. 4.

### Generation of mutant *S. argillaceus* ΔL

To this aim, plasmid p∆L (Table 2) was generated as described in Additional file 1. This construct contains most of the *mtmL* gene replaced by the apramycin cassette in the same direction of transcription. The plasmid p∆L was used to transform *S. argillaceus* protoplasts. Transformants were selected with apramycin and tested for their susceptibility to thiostrepton. PCR analysis and sequencing using primers LF and Q (Additional file 1: Figure S3) were performed to confirm the replacement of the wild type copy of *mtmL* by the mutated one (Additional file 1: Figure S3).

### Metabolite analysis, purification and structure elucidation

Extraction and spectroscopic analyses of metabolites produced by *S. argillaceus* strains were carried out as described [23]. For the UHPLC analyses of *S. albus* metabolites, samples were eluted with 10% acetonitrile during 1 min, followed by a linear gradient from 10% to 100% acetonitrile in 7 min, at a flow rate of 0.5 mL/min and a column temperature of 35 °C. Production and purification of SEK15 **1** and 2-hydroxy nogalonic acid **2** was carried out by growing *S. argillaceus* ∆L in 1L of R5A solid medium. Plates were inoculated with 25 µl of spores (10^6^ spores/mL), incubated for 7 days at 30 °C and extracted with 1:1 volume of ethyl acetate. The organic extract was evaporated under vacuum and purified by semi-preparative HPLC using an Agilent 1200 instrument fitted with a C-18 column (100 × 21 mm, 5 μm, Agilent). The following elution profile with a flow rate of 9 mL/min was applied: an isocratic condition of 5% acetonitrile for 5 min, followed by 45 min linear gradient to 100% acetonitrile. The purified samples were analyzed by high resolution UHPLC-MS on a Bruker MaXis Impact electrospray ionization time-of-flight mass spectrometer (ESI-TOF-MS) connected to a Dionex 3000 RS UHPLC instrument with UV absorption detected at 280 nm and fitted with an Agilent Zorbax Eclipse Plus C18 column (100 × 2.1 mm, 1.8 μm, 25  °C) at a flow rate of 0.2 mL/min using the following elution program: 0 min, 5% ACN/95% H_2_O; 12 min, 100% ACN; 17 min 100% CAN (both ACN and H_2_O contained 0.1% formic acid). The NMR spectra were recorded at 25  °C in DMSO-d6 for SEK15 and CD_3_OD for 2-hydroxy nogalonic acid on a Bruker Avance II 700 MHz spectrometer. Structure elucidation of **2** was carried out using a combination of 1D and 2D NMR (1H, 13C, COSY, HSQC and HMBC) and high resolution MS/MS experiments (Additional file 3). Briefly, H8 and H10 appear as doublets at 7.15 and 6.65 ppm respectively, all the rest of the proton signals appear as singlets. The connectivity of the structures was mainly established using HMBC correlations. Key correlations observed include: H2 to C4, C5 and C16; H4 to C2, C3, C5, C6, C14 and C16, and weak correlation to C15; H18 to C16, C17, C19 and C20.

## Supplementary information

**Additional file 1:** Overexpression/deletion of *mtmL*. Generation of plasmid pΔL. Generation of plasmid pDZL10. **Figure S1.** Comparison of MtmL with putative Acyl-CoA Ligases. **Figure S2.** Production of MTM by strains overexpressing *mtmL*. **Figure S3.** Generation and analysis of mutant *S. argillaceus* ΔL.

**Additional file 2:** Chemical characterization of SEK15. **Table S1.** NMR assignment.

**Additional file 3**: Chemical characterization of 2-hydroxy nogalonic acid. **Table S2.** NMR assignment. **Figure S4.** Structure and key HMBC correlations. **Figure S5.** High resolution MS.

**Additional file 4:** Generation of gene cassette plasmids for 4DMPC. **Table S3.** Primers used in this work.
