# Supplementary material for: Heterologous reconstitution of the biosynthesis pathway for 4-demethyl-premithramycinone, the aglycon of antitumor polyketide mithramycin
Source: Microb Cell Fact. 2020 May 24;19:111. doi: 10.1186/s12934-020-01368-3 (PMC7247220; doi:10.1186/s12934-020-01368-3)

Additional file 1. Overexpression/deletion of *mtmL*

*Generation of plasmid p*ΔL: In order to generate the mutant strain *S. argillaceus* ΔL, the plasmid pΔL was constructed as follows. A 10 Kb BamHI DNA fragment containing *mtmD* to *mtmP* genes and including *mtmL*, was isolated from cosmid cosAR7 and subcloned into the vector pBSKT previously digested with BamHI, generating the construct pDZ4. From this, a BglII-SacI DNA fragment containing the 5'-end of *mtmL*, *mtmQ*, *mtmX* and the 5'-end of *mtmP* was subcloned into pUK21 previously digested with the same restriction enzymes, generating pDZL1. In the following step, a PstI-BglII DNA fragment from pDZ4 containing the 3'-end of *mtmD*, *mtmE*, *mtmTII*, *mtmOII*, *mtmOIII*, and the 3'-end of *mtmL* was subcloned into pUK21 previously digested with PstI-BamHI to generate the construct pDZL2. Then, an apramycin resistance cassette isolated from pEFBA was subcloned as a HindIII-NdeI DNA fragment into the plasmid pDZL1, previously digested with the same restriction enzymes, generating the construct pDZL3. The next step was subcloning the whole insert from pDZL2 as a BglII-XbaI fragment into the same sites of pDZL3 downstream of the apramycin resistance cassete, to generate pDZL4. Finally, the whole insert in pDZL4 was rescued as a SpeI DNA fragment and subcloned into the same sites of pBSKT, which is a suicide plasmid in *Streptomyces*, to generate the final plasmid pΔL.

Generation of plasmid pDZL10: This plasmid was constructed to overexpress *mtmL* into *S. argillaceus* wild type strain and to complement *S. argillaceus* ΔL mutant. To generate pDZL10 the gene *mtmL* was subcloned from plasmid pDZ4 as a BclI-EcoRI fragment into the vector pIJ2925 previously digested as BamHI-EcoRI to obtain the construct pDZL6. From this plasmid, *mtmL* was subcloned as an XbaI-EcoRI fragment into the same sites of pEM4, generating the construct pDZL7. Finally, the *mtmL* gene was subcloned from pDZL7 as a BamHI-EcoRI fragment into the multicopy, conjugative, bifunctional *Streptomyces*- *E.coli* vector pEM4T, downstream of the erythromycin resistance gene promoter (*ermEp**) to generate the final plasmid pDZL10.

Figure S1. Comparison of MtmL with putative Acyl-CoA Ligases

```
ADE34493        mattdltpladgtalgepletiagl--iggprvdqlvtraaqrapdrvavrsatvt----  54
ACN64850        -------------------------mrepverldelprlaaeahpdrvalrtdrsd----  31
CAE17553        --------------------mpfdlarvpevpldgllrraaaaapdrvavrtptgt----  36
WP_053692533    ----------------------mprfaypelpldgllrraavrdpagrairtatgagftt  38
WP_031013729    -------------------------mpvpavpldgllrraaarspgrvavrdengt----  31
WP_093909794    -------------------------msvpalpldgllrraaeraphhpairderge----  31
SDP01439        ---------------------msealsvpalpldgllrraaeraphhpairderge----  35
WP_051703560    ---------------------mseapsvpavpldgllrraaenfpervavrdehga----  35
MtmL            ---------------------mselppgpavpldgllrlaaerhpeqvavrderga----  35
WP_164189986    ------------------------mpsgpavpldellrraaerhpdrvavrderga----  32
                                                :* *   **   *    *:*

ADE34493        -vtyaeleaqtdraaamlqglvdgpgavvaiaglmdpefaslffgisragavsavlnpfl  113
ACN64850        altfgeleqrvsgcaariralvggpgttvaitsvlhpdfavayygtlraghvvvpvnpfl  91
CAE17553        -ttyaeldgraddcarslsraaggsgtavglvasldpsfavayygavradhvvvlinphl  95
WP_053692533    tvtfaeldgradrfasylehtlgrrgarvgvanvldpvfaaayygtvrsgntvvlvnpli  98
WP_031013729    -htyagldayadrfaralrrrtggaatavgvasvldavfaagfygtsrsgnrvvmvnplm  90
WP_093909794    -tdyagldayadrfaralrrtvgadgaavgvasvldtvfaaafygtsrsgnrivlvnplv  90
SDP01439        -tdyagldayadrfaralrrtvgadgaavgvasvldtvfaaafygtsrsgnrivlvnplv  94
WP_051703560    -igyaeldayadrfalallrltrgrqtavgvagvldpvfaagfygtsrsgnrvvmvnpli  94
MtmL            -vdyagldaradrfarallrltggrptavgvasvldpvfaaafygtsrsgnrvvlvnplv  94
WP_164189986    -tnyarldayadecarallrltggrrtavgvasvldqvfaaafygaarsgnrivmvnplv  91
                   :. *:  ..  *  :        : *.:.  :.  **  ::*  *:.   . :** :

ADE34493        paerlahilatsgarvavlspemyqriesvrgrvpqletviltrrdagl---ddvptlae  170
ACN64850        rakglealvaasgarlvltteeiaaelg---dtvpsldi-------------gdrt---  131
CAE17553        rgdalahvlsvagttvvvappefaervaqvrgklphlrdvlspdvlvqgragpggr----  151
WP_053692533    keaglvhvlrtaeaevafvptataellvkvgdrlphlrsvvvtdap-dgvvpgdalplaa  157
WP_031013729    reavlahvfrtagieialvgaetagrlaavrddlpalrevyvvdaa-pgelpggarplae  149
WP_093909794    reaalehvfrtaavevavvpaetarrlatvrdrlpdlrevyvvdadredrlpegtrpvad  150
SDP01439        reaalehvfrtaavevavvpaetarrlatvrdrlpdlrevyvvdadredrlpegtrpvad  154
WP_051703560    reaalehvfrtagveialvpvetavrlqtvrgylpalrevfvvdadravtlpkctrplad  154
MtmL            repvlehvfrtagieialvsaetrsp-ggrraalpdlrevyvvdadrtgppppgtrpldd  153
WP_164189986    repvlehvfgtagieialvsaetaarlatvranlpalrevyvvdadrvgplpegtrplad  151
                    *  :. .:   :..               :* *

ADE34493        qlaaap--aapdlsgvgddpdalaclqftsgttgsakacqlshrnltvnaaqtvyaqtvd  228
ACN64850        --lw---dtgplreapavrpddvasihftsgttgapkgvrqthhnlavnaaqtvhahgld  186
CAE17553        ---------grdapgsaphldsvacvqftsgttgepkavqlthrnlvvnaaqiasvhgld  202
WP_053692533    alaaape---ravlpgavdldadvciqfttgttglpkgvrlthrnlvanaaqtaaahdlg  214
WP_031013729    lfaddgpdegpvaepagrdldavacvqftsgttgppkgvqlthrnlvtnaaqvahvlrld  209
WP_093909794    llaaaedgpp-lppgpdrdladvvcvqftsgttgppkgvqlthrnlvanaaqaayalgld  209
SDP01439        llaaaedgpp-lppgpdrdladvvcvqftsgttgppkgvqlthrnlvanaaqaayalgld  213
WP_051703560    llgwdgpsaaalpdpaavdpdsvacvqftsgttgppkgvqlthrnlvtnaaqaahvlgld  214
MtmL            lltedgta--alpdpagvdldsvvcvqftsgttgppkgvrlthrnlvanaaqaahalgld  211
WP_164189986    llagggpaaptlpgpagveldsvvcvqftsgttgppkgvqlthhnlvanaaqaahalgld  211
                                       ..::**:****  *. : :*:**..**** . .  :.

ADE34493        ewsvvfnclptfhlmhltlavtavathvlwpgsdpvasiraadrlrathyyslpvrlmhl  288
ACN64850        dssvtvnhlptfhpmhlnsalfaaaeqvlatdpdlaaslrtanearatryyslpmrlsrl  246
CAE17553        gdavtlnnlplyhpmhlnaavyagatqvlcpspdpaeaieaanrhrathyyslpvrlahl  262
WP_053692533    sdsvtlnhlplyhtmhlnsgvyagacqvlctdpdpvaslaasaraeathyyglparlhrl  274
WP_031013729    esavclnhlplyhlmhlnatvyaggtqvlchdpdpvasfavasaerathyfglpvrlarl  269
WP_093909794    tdavclnhlplyhlmhlnatvyaggtqvlchdpdpvesfaaavraeathyfglpvrlara  269
SDP01439        tdavclnhlplyhlmhlnatvyaggtqvlchdpdpvesfaaavraeathyfglpvrlara  273
WP_051703560    adcvclnhlplyhlmhlnaavlagatqvlchdpdpvasfataadagathyfglpvrltrl  274
MtmL            agsvclnhlplyhvmhldsavyagatqvlchdpdpvasvaaaaeagathyfglpvrlarl  271
WP_164189986    adsvclnhlplyhlmhlnaavyagatqvlchdpdpvasfaaavdtgathyfglparltrl  271
                  .* .* ** :* ***   : * . :**  . * . :. .:    **:*:.** ** :

ADE34493        aadpelsqle--apalraifsggsslpkpatvalaqqfgvpvvqgyglaetspshhlgsp  346
ACN64850        aarpdlselr--ldtveavfsggsalavpaarrlgdhfgipvlqgyglaetapmthcgsr  304
CAE17553        aadprlpglr--letvravfsggsallpaqartlgthfgipvvqgyglaetsplthgepp  320
WP_053692533    aqderlthdfpagrrltavlsggtalaptaartlrarlgvpviqgyglaelsplshcqdp  334
WP_031013729    agdprfagvt-agprlrvvasggsalapavagalrerlgvpviqgyglaelsplshndlp  328
WP_093909794    aaderfgrav-agprlrlvasggsalapavaealrerlgvpviqgyglaelsplshndrp  328
SDP01439        aaderfgrav-agprlrlvasggsalapavaealrerlgvpviqgyglaelsplshndrp  332
WP_051703560    aadqrlaavv-pgpklrliasggsalaplvadrlreqlgvpviqgyglaelsplshndrp  333
MtmL            aadprlasvv-pgpelglvrsggsrlapavaarlrerlgvpviqgyglaelsplshndrp  330
WP_164189986    aadqrlasvv-pgsglrlvasggsalapavaarlrnqlgvpviqgyglaelsplshndrp  330
                *    :         :  :  ***: *    :  *  ::*:**:******* :*  *

ADE34493        hrpkpgssgqpvpgaetrivdvdtgkplpvggkgeiqvrgpqlmlgylgrtigqdtdeeg  406
ACN64850        trpkegscgppvprtecrivdlatgkalgpgergeiqvrgpqlmkgylgepdggaldaeg  364
CAE17553        arprpgsvgpevpgtecrvvdmdrrtplpagrdgeiqvrgpqlmrgylgaa-gpavdadg  379
WP_053692533    hrpksgsvgpavpgtecrivavdtstpldvyatgevqvrgpqvmagylddsapspidadg  394
WP_031013729    drsrpgsvgpavpgtecrivdletgedvaagekgevlvrgpqlmagylglpdapdvdadg  388
WP_093909794    ghskpgsvgpavpgtecrivdldtgatadagqkgevllrgpqlmagylglpdapdidadg  388
SDP01439        ghskpgsvgpavpgtecrivdldtgatadagqkgevllrgpqlmagylglpdapdidadg  392
WP_051703560    erskpgsvgpvvpgtecrivdletgadlgtgrpgevllhgpqlmagylnlpdapdidtdg  393
MtmL            erskpgsvgpavpgtecrivdletgaaldpgrpgevllrgpqlmagylglpdapdidaag  390
WP_164189986    erskpgsvgpavpgtecrvvdletgsdlgpghpgevllrgpqlmtgylglpdaadidaag  390
                 : : ** *  ** :* *:* :           **: ::***:* ***.       *  *

ADE34493        wfstgdvgtlddegflfwidrikdtfkcdnwlvspteiervllrhpavadctvlgipdal  466
ACN64850        wfatgdvgevdeddclfvvdrlkdvfkcdnwlvspqeieqvllahpavadcavvdhpdef  424
CAE17553        wfstgdighrdqdgylylvdrikdvfkyenwlvspteieqvlithpavrdcavvdhpepf  439
WP_053692533    wfstgdvgycnedgvlflvdrlddvfkydnelvspsgvedvigadprvaecivagwpdpv  454
WP_031013729    wlhtgdvgyqdadgwlflvdrikdvfkvdnelvspseiedvlrrdpevadcvvvdqadas  448
WP_093909794    wfhtgdvgyedrdgwlfltdrikdvfkvdnelvspteierllaadadvadcvvvdhpdef  448
SDP01439        wfhtgdvgyedrdgwlfltdrikdvfkvdnelvspteierllaadadvadcvvvdhpdef  452
WP_051703560    wfhtgdvgyqdgdgwlflvdrikdvfkvdnelvspseierilmrdgdiadcvvadmpdef  453
MtmL            wfhtgdvgyqdedgwlflvdrikdvfkvdnelvspseieqvllqdpdvadcvvadlpdef  450
WP_164189986    wfhtgdvgyqdedgwlflvdrikdvfkvdnelvspseierillhdadvadcvvvdipdef  450
                *: ***:*  : :. *:  **:.*.** :* **** :* ::  .  : :* * .  :

ADE34493        rgsvacavivsrdpav-----tvedfaefvnpkvpyyeelarvvlveaiarsatgkverr  521
ACN64850        sgavavalvvtrrpeepe---aldgiaadvnsglpyyqhirhiravpdiarsgggkvrrq  481
CAE17553        sgavahafvvldedaa---lpgpaelaahvndqvpyyqqikyldvvervprspngkilrr  496
WP_053692533    hgalvwtgivlrdptapglldvldsiveranarlndfeqirrvevldavprspvgkperr  514
WP_031013729    rsavvwagvvtrsadp----dvlaavaaranaqladhqrirgierldavprspngkverr  504
WP_093909794    sgavvwagvvpsgdgp----vdlaavvarvnaqladhqrirhvepltgvprspngkierr  504
SDP01439        sgavvwagvvpsgdgp----vdlaavvarvnaqladhqrirhvepltgvprspngkierr  508
WP_051703560    sgaavwagivpassgp----vdlravveranaqladhqrirraeqltavprspngkaerr  509
MtmL            sgavvwagvvpagdgp----vdlnpivaranallsdhqrirraerltavprspngktert  506
WP_164189986    sgavvwagivpvgdrp----vdlrlvvaranaqlsehqwirraerlkavprspngkaerr  506
                 .: . : :*               ..  .*   :  .: :     :  : **  **  *

ADE34493        llrdkvvagelg----------------------  533
ACN64850        dlrrlftelfatrg--------------------  495
CAE17553        elraraahtdra--drlhpgaagrphsdligenp  528
WP_053692533    alrlslrgraaaeaaav-----------------  531
WP_031013729    pirerlrasval----------------------  516
WP_093909794    rlrerlrgtaaa----------------------  516
SDP01439        rlrerlrgtaaa----------------------  520
WP_051703560    llreqlraeppspltsrrpcrpgepsw-------  536
MtmL            rlrerlra--------------------------  514
WP_164189986    slrerlradaa-----------------------  517
                 :*
```

CAE17553 (CmmLII); ACN64850 (PokL); ADE34493 (SsfL2)

Figure S2. Mithramycin production in R5A solid cultures by *Streptomyces argillaceus* pEM4T (control) and *S. argillaceus* pDZL10

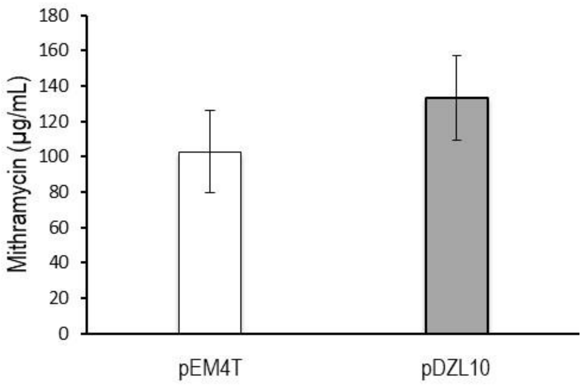

Figure S3. Generation and analysis of mutant *S. argillaceus* ΔL. (A) Scheme representing the replacement event for generation of mutant ΔL. WT, wild type strain; *aac(3)IV*, apramycin resistance gene; (B) PCR analysis of mutant ΔL. PCR products from the wild type (WT) strain and from mutant ΔL, using oligonucleotides Q (ACAGCCAGCAGTACTCCGT) and LF (ATGCGCTGGTGGCGGAGAGCA).λ, PstI-digested Lambda DNA.

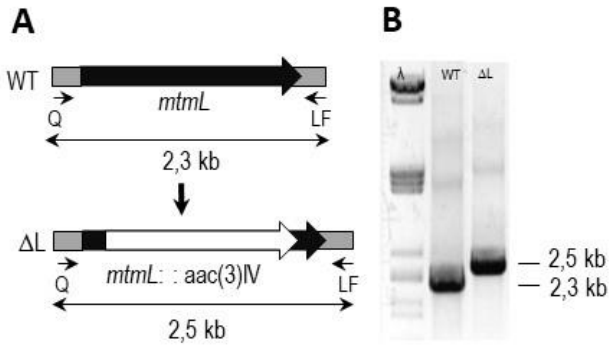

Supplement: Supplementary file 1 — Additional file 1: Overexpression/deletion of mtmL. Generation of plasmid pΔL. Generation of plasmid pDZL10. Figure S1. Comparison of MtmL with putative Acyl-CoA Ligases. Figure S2. Production of MTM by strains overexpressing mtmL. Figure S3. Generation and analysis of mutant S. argillaceus ΔL. [file 12934_2020_1368_MOESM1_ESM.pdf]
