# Supplementary material for: Heterologous reconstitution of the biosynthesis pathway for 4-demethyl-premithramycinone, the aglycon of antitumor polyketide mithramycin
Source: Microb Cell Fact. 2020 May 24;19:111. doi: 10.1186/s12934-020-01368-3 (PMC7247220; doi:10.1186/s12934-020-01368-3)
Supplement: Supplementary file 2 — Additional file 2: Chemical characterization of SEK15. Table S1. NMR assignment. [file 12934_2020_1368_MOESM2_ESM.pdf]

Additional file 2. Chemical characterization of SEK15

Table S1. NMR assignment of SEK15 (solvent DMSO-d6, 700MHz)

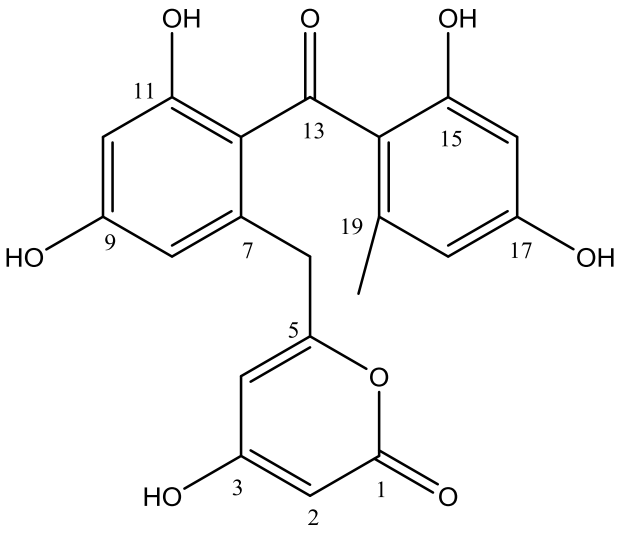

Carbon	1H (δ, ppm, J,)	13C (ppm)	Key HMBC
1		164.0	
2	5.14 (d, 2Hz)	88.4	C1, C3, C4
3		170.7	
4	5.69 (d, 2Hz)	100.6	C2, C3, C5, C6
5		163.4	
6	3.52 (s, b)	36.4	C4, C5, C7, C8, C12
7		135.2	
8	6.20 (d, 2Hz)	109.4	C6, C9, C10, C12, C13
9		159.7	
10	6.24 (d, 2Hz)	101.3	C8, C9, C11, C12, C13
11		157.2	
12		121.0	
13		200.2	
14		117.2	
15	12.20 (15-OH, s)	163.1	
16	6.12 (d, 2Hz)	100.5	C13, C14, C15, C17, C18
17		162.2	
18	6.08 (d, 2Hz)	110.7	C13, C14, C16, C17, C20
19		141.4	
20	1.86 (s)	20.5	C14, C15, C16, C18, C19
