# Supplementary material for: Heterologous reconstitution of the biosynthesis pathway for 4-demethyl-premithramycinone, the aglycon of antitumor polyketide mithramycin
Source: Microb Cell Fact. 2020 May 24;19:111. doi: 10.1186/s12934-020-01368-3 (PMC7247220; doi:10.1186/s12934-020-01368-3)

Additional file 3. Chemical characterization of 2-hydroxy nogalonic acid

Table S2. NMR assignment for 2-hydroxy nogalonic acid (solvent CD_3OD)

C position	^{1}H (ppm)	^{13}C (ppm)*	Key HMBC
1		171.3	
2	3.77 (s)	38.9	C1, C3, C4, C16
3		142.0	
4	7.66 (s)	121.8	C2, C3, C5, C6, C14, C15, C16
5		124.6	
6		181.0	
7		135.2	
8	7.15 (s)	108.8	C6, C10, C12
9		165.8	
10	6.65 (s)	107.8	C7, C8, C12, C13
11		164.7	
12		108.9	
13		189.1	
14		114.5	
15		160.3	
16		132.0	
17		199.7	
18	4.16 (s)	57.8	C16, C17, C19, C20
19		202.2	
20	2.22 (s)	30.7	C18, C19

* From HSQC and HMBC

Figure S4. Structure and key HMBC correlations for 2-hydroxy nogalonic acid

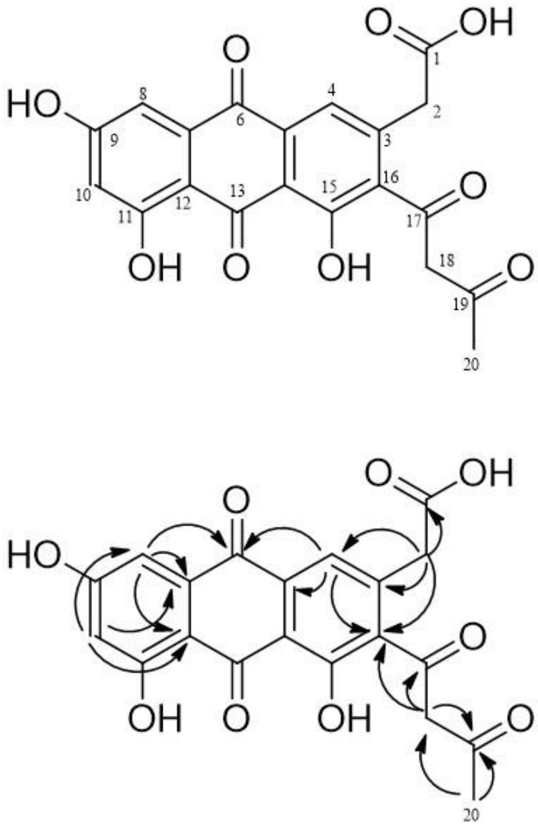

Figure S5. High resolution MS of 2-hydroxy nogalonic acid

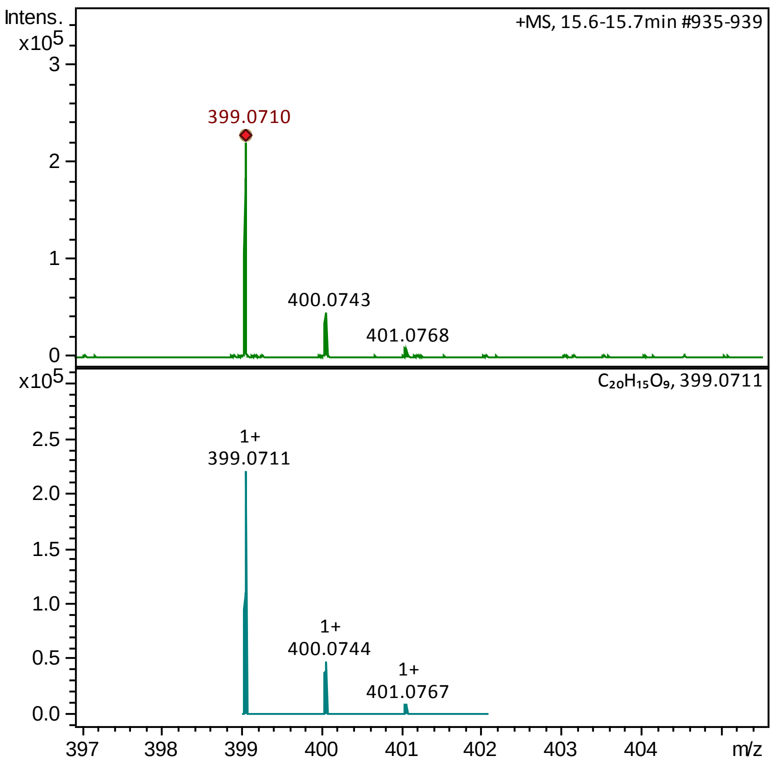

Supplement: Supplementary file 3 — Additional file 3: Chemical characterization of 2-hydroxy nogalonic acid. Table S2. NMR assignment. Figure S4. Structure and key HMBC correlations. Figure S5. High resolution MS. [file 12934_2020_1368_MOESM3_ESM.pdf]
