# Supplementary material for: Heterologous reconstitution of the biosynthesis pathway for 4-demethyl-premithramycinone, the aglycon of antitumor polyketide mithramycin
Source: Microb Cell Fact. 2020 May 24;19:111. doi: 10.1186/s12934-020-01368-3 (PMC7247220; doi:10.1186/s12934-020-01368-3)
Supplement: Supplementary file 4 — Additional file 4: Generation of gene cassette plasmids for 4DMPC. Table S3. Primers used in this work. [file 12934_2020_1368_MOESM4_ESM.pdf]

Additional file 4. Generation of gene cassette plasmids for 4DMPC

A cloning strategy was developed to allow the expression of different gene cassettes involved in early stages of MTM biosynthesis. The different genes (*mtmPKS, mtmL, mtmQ, mtmX, mtmY, mtmOII, mtmTI* y *mtmTII*) were amplified flanked by unique restriction sites using the oligonucleotides shown in Table S3. To facilitate the following cloning steps, the PCR products were individually subcloned into the vector pCR-blunt to generate plasmids pbluntPKS, pbluntL, pbluntQ, pbluntX, pbluntY, pbluntOII, pbluntTI and pbluntTII, respectively. The shuttle bifunctional *E. coli-Streptomyces* vector pEM4 was used to express genes under the erythromycin resistance gene promoter (*permE**), as follows:

<u>pDZPKS1</u>: a SpeI-EcoRI fragment containing *mtmP*, *mtmK* and *mtmS* was subcloned from pbluntPKS into the XbaI and EcoRI sites of pEM4, generating pDZPKS. Then, to facilitate following cloning steps and allow the expression of further genes, a second *permE** promoter was introduced downstream of *mtmS*. To do this, the HindIII-EcoRI (blunt ended) fragment containing *mtmP mtmK, mtmS* and the *permE** promoter was subcloned from pDZPKS into the HindIII (blunt ended) site of pEM4 upstream of its *permE** promoter.

<u>pDZPKSQ</u>: *mtmQ* was rescued as a NheI-XbaI DNA fragment from pbluntQ and subcloned in the right orientation, into the XbaI site of pDZPKS1.

<u>pDZPKS5:</u> first, *mtmL* was isolated as a SpeI-XbaI DNA fragment from pbluntL and subcloned in the right orientation, into the XbaI site of pDZPKS1 generating pDZPKS2. Then, *mtmQ* was rescued as a NheI-XbaI DNA fragment from pbluntQ and subcloned in the right orientation, into the XbaI site of pDZPKS2 generating pDZPKS3. Afterwards, *mtmX* was digested as a Spe-XbaI DNA fragment from pbluntX and subcloned in the right orientation, into the same sites of pDZPKS3 generating pDZPKS4. Finally, *mtmY* was rescued as HpaI-XbaI from plasmid pbluntY and subcloned in the same restriction sites pDZPKS4 generating the final construct pDZPKS5.

pDZPKS8: *mtmY* was isolated from the plasmid pbluntY as a SpeI-XbaI DNA fragment and subcloned in the same sites of pDZPKS3.

pDZPKS9: *mtmQ* and *mtmY* were isolated as a NheI-XbaI DNA fragment from pDZPKS8 and subcloned in the right orientation, into the XbaI site of pDZPKS1.

pDZPKS10: *mtmTI* was isolated as a SpeI-XbaI DNA fragment from pbluntTI and subcloned in the right orientation, into the XbaI site of pDZPKS9.

pDZPKS11: *mtmX* was isolated from pbluntX as a SpeI-XbaI DNA fragment and subcloned in the right orientation, into the site XbaI of pDZPKS9.

pDZPKS12: First, *mtmOII* was isolated as a PacI-XbaI DNA fragment from pbluntOII and subcloned in the same restriction sites of pDZPKS5, generating pDZPKS6. Then, *mtmTI* was rescued as a SpeI-XbaI DNA fragment from pbluntTI and subcloned in the right orientation, into the XbaI site of pDZPKS6 generating pDZPKS7. Afterwards, *mtmTII* was rescued as a SpeI-XbaI DNA fragment from pbluntTII and subcloned in the right orientation, into the site XbaI of pDZPKS7 generating pDZPKS14. Finally, *mtmQ; mtmX; mtmY; mtmOII; mtmTI* and *mtmTII* were rescued as a NheI-XbaI DNA fragment from pDZPKS14 and subcloned in the right orientation, into the XbaI site of pDZPKS1 generating the final construct pDZPKS12.

pDZPKS13: *mtmTII* was isolated as a SpeI-XbaI fragment from the vector pbluntTII and subcloned into a XbaI site of pbluntOII to generate pbluntOIITII. Then, *mtmOII* and *mtmTII* were isolated as a SpeI-XbaI DNA fragment and subcloned in the site XbaI of pDZPKS11 generating pDZPKS13.

pDZPKS15: *mtmOII* was isolated as a BamHI-EcoRI DNA fragment from the vector pbluntOII and subcloned in the same site of pEM4AT.

pDZPKS21: *mtmTI* was digested as a EcoRI DNA fragment from pblunTI and subcloned in the right orientation, into the same site of pDZPKS15 generating pDZPKS19. Then, *mtmTII* was

rescued as a BamHI-NheI fragment from pblunTII and subcloned in the sites BamHI-SpeI of pDZPSK19.

Table S3. Primers used in this work.

Cebador	Secuencia (5´→ 3´)
PKS-AM F	AGACTAGTCATGAGCAGGGGGACAAC (SpeI)
PKS-AM R	AGGAATTCGTCACGGTTCGGCTCCTC (EcoRI)
L-AM F	AGTCTAGAGCTAGCGTCACCACGATGGTTCTCCT (XbaI-NheI)
L-AM R	AGACTAGTCCTAGGCCAACAGTGTCAGCAACCTG (SpeI-AvrII)
Q-AM F	AGTCTAGAACTAGTCGTCGAGTCCGGCGTAGT (XbaI-SpeI)
Q-AM R	AGGCTAGCATCCAGCCGGAGTCCAAT (NheI)
X-AM F	AGACTAGTCTCGAGGCGCATTGGACT (SpeI)
X-AM R	AGTCTAGAGTTAACAGACACCGGGAGGAGGTG (XbaI-HpaI)
Y-AM F	AGGTTAACCGTACCGATCTGTTCACCAC (HpaI)
Y-AM R	AGTCTAGATTAATTAACTTACACGACGGCAACAAGA (XbaI-PacI)
OII-AM F	AGTCTAGACCTGTCAGTCCCATGTCCA (XbaI)
OII-AM R	AGTTAATTAACCCGACGTGTTCATCGAG (PacI)
TI-AM F	AGACTAGTACGGCGTCGTCCTCGACGA (SpeI)
TI-AM F2	AGTTAATTAATAACGGCGTCGTCCTCGACGA (PacI)
TI-AM R	AGTCTAGAACGCAGACGTCGGTGTGCA (XbaI)
TII AM F	AGTCTAGAACGGCTGACCATCCGTCGGCAT (XbaI)
TII AM R	AGACTAGTTCTGCGCGACGAACAGCACGT (SpeI)
